# Machine Learning Analysis of Time-Dependent Features for Predicting Adverse Events During Hemodialysis Therapy: Model Development and Validation Study

**DOI:** 10.2196/27098

**Published:** 2021-09-07

**Authors:** Yi-Shiuan Liu, Chih-Yu Yang, Ping-Fang Chiu, Hui-Chu Lin, Chung-Chuan Lo, Alan Szu-Han Lai, Chia-Chu Chang, Oscar Kuang-Sheng Lee

**Affiliations:** 1 Institute of Clinical Medicine National Yang Ming Chiao Tung University School of Medicine Taipei Taiwan; 2 Stem Cell Research Center National Yang Ming Chiao Tung University Taipei Taiwan; 3 Department of Physiology and Pharmacology Chang Gung University College of Medicine Taoyuan Taiwan; 4 Department of Plastic and Reconstructive Surgery Chang Gung Memorial Hospital Taoyuan Taiwan; 5 Division of Nephrology Department of Medicine Taipei Veterans General Hospital Taipei Taiwan; 6 Center for Intelligent Drug Systems and Smart Bio-devices Hsinchu Taiwan; 7 Division of Nephrology Department of Medicine Changhua Christian Hospital Changhua Taiwan; 8 Institute of Systems Neuroscience National Tsing Hua University Hsinchu Taiwan; 9 Department of Medicine Kuang Tien General Hospital Taichung Taiwan; 10 Department of Nutrition Hungkuang University Taichung Taiwan; 11 Department of Orthopedics China Medical University Hospital Taichung Taiwan

**Keywords:** hemodialysis, intradialytic adverse events, prediction algorithm, machine learning

## Abstract

**Background:**

Hemodialysis (HD) therapy is an indispensable tool used in critical care management. Patients undergoing HD are at risk for intradialytic adverse events, ranging from muscle cramps to cardiac arrest. So far, there is no effective HD device–integrated algorithm to assist medical staff in response to these adverse events a step earlier during HD.

**Objective:**

We aimed to develop machine learning algorithms to predict intradialytic adverse events in an unbiased manner.

**Methods:**

Three-month dialysis and physiological time-series data were collected from all patients who underwent maintenance HD therapy at a tertiary care referral center. Dialysis data were collected automatically by HD devices, and physiological data were recorded by medical staff. Intradialytic adverse events were documented by medical staff according to patient complaints. Features extracted from the time series data sets by linear and differential analyses were used for machine learning to predict adverse events during HD.

**Results:**

Time series dialysis data were collected during the 4-hour HD session in 108 patients who underwent maintenance HD therapy. There were a total of 4221 HD sessions, 406 of which involved at least one intradialytic adverse event. Models were built by classification algorithms and evaluated by four-fold cross-validation. The developed algorithm predicted overall intradialytic adverse events, with an area under the curve (AUC) of 0.83, sensitivity of 0.53, and specificity of 0.96. The algorithm also predicted muscle cramps, with an AUC of 0.85, and blood pressure elevation, with an AUC of 0.93. In addition, the model built based on ultrafiltration-unrelated features predicted all types of adverse events, with an AUC of 0.81, indicating that ultrafiltration-unrelated factors also contribute to the onset of adverse events.

**Conclusions:**

Our results demonstrated that algorithms combining linear and differential analyses with two-class classification machine learning can predict intradialytic adverse events in quasi-real time with high AUCs. Such a methodology implemented with local cloud computation and real-time optimization by personalized HD data could warn clinicians to take timely actions in advance.

## Introduction

Hemodialysis (HD) therapy has a substantial role in critical care management [1]. Due to oliguria or even anuria, most patients with renal failure require fluid removal during HD therapy to maintain an euvolemic status. The volume-dependent component of hypertension may be corrected by fluid removal, but the ultrafiltration process exposes HD patients to the risks of hemodynamic instability, which may lead to fatal consequences such as cardiac arrest [2]. Intradialytic hypotension is the most frequent complication during HD [3-7] and has been identified as a pivotal cause of reduced HD efficacy [4,8]. Acutely, intradialytic adverse events can be fatal; chronically, frequent intradialytic adverse events increase patient morbidity and long-term all-cause mortality [3,9,10]. Therefore, it is urged to develop a solution for this unmet medical need.

The Crit-Line (Fresenius Medical Care) monitor is a device developed to assist with fluid removal during ultrafiltration by noninvasively monitoring real-time hematocrit, oxygen saturation, and intradialytic volume status, using an optical transmission method [11]. Although uncontrolled studies have suggested that this device reduced intradialytic symptoms [5,12] and assisted in the assessment of target weight [13,14], an unblinded randomized controlled trial showed a higher hospitalization rate in the Crit-Line group than in the control group [15]. Therefore, novel solutions are urged to solve this unmet medical need.

Artificial intelligence has been applied to HD patients to assist clinical practice, including prediction of urea clearance [16-19], dietary protein intake [17,20], volume status [21], erythropoiesis-stimulating agent response [22-26], iron supplement response [22,24], hemoglobin level [27], HD quality [28-31], mortality [32], etc. Although artificial intelligence has also been applied to predict intradialytic hypotension risks, previous studies lack time-series data input [17,33]. Therefore, we aimed to include time-series data in a machine learning model to predict intradialytic adverse events. Herein, we hypothesize that the machine learning method can predict intradialytic adverse events in an unbiased manner. To solve this unmet need, we conducted this study.

## Methods

### Study Protocol and Subjects

This was a retrospective observational study in a single institution. We reviewed the records of all patients who underwent maintenance HD therapy at Changhua Christian Hospital, a tertiary-care referral center in middle Taiwan, between August 2017 and October 2017. During this period, 129 patients were eligible for enrollment evaluation, and 108 patients completed the 3-month study. HD sessions were excluded for the following three reasons: (1) session interruption due to dialyzer exchange, (2) more than one interruption per session due to patient urination or defecation, and (3) inability of patients to freely express their discomfort during the session. Eventually, a total of 4221 HD sessions from 108 patients were used to build the model. Each patient received either 39 or 40 HD sessions during the 3-month study period.

The Institutional Review Board of our institution approved all protocols in April 2017 before the study began, and the protocols conformed to the ethical guidelines of the Helsinki declaration. The need for informed consent was waived because of the retrospective nature of the study.

### Dialysis and Physiological Data Collection

Demographic information from medical records, including age, gender, and years under dialysis treatment, were included for model building. Dialysis and physiological data of the enrolled patients during the 4-hour HD session were included in the study. Physiological data were measured and recorded by medical staff every 30 to 60 minutes approximately. Dialysis data were collected from the dialysis machine automatically. Intradialytic adverse events were documented by medical staff according to physiological measurements or patient complaints, as shown in Table 1. The list of HD machine readouts is presented in Multimedia Appendix 1.

For each HD session i (i=1-4221), the data set HD_i_ consisted of records {Y_j,k_, T_k_}, where j (range 1-9) is the index for the dialysis and physiological measurements, and k is the index of time when a measurement is taking. Y_j,k_ is the value of the measurement j at time T_k_. HD_i_ also included additional time-invariant patient-specific information Y_j_ (j=10-13), including age, gender, years under dialysis treatment, and predialytic weight (Multimedia Appendix 2). According to the manufacturer default setting, the machine-derived dialysis data are recorded from the dialysis machine automatically once the value of venous pressure or transmembranous pressure alters and becomes different from the last measurement at T=T_k-1_. Therefore, the time interval T_k_ − T_k-1_ between any two consecutive records may not be equal.

**Table 1:** List of intradialytic adverse events.

Adverse event	Episodes, n
Muscle cramps	138
Blood pressure elevation	108
Low blood pressure	64
Miscellaneous	45
Headache	28
Lightheadedness	26
Chest tightness	23
Vascular access thrombosis	23
Cold sweating	22
Nausea/vomiting	12
Fever	10
Tachycardia	10
Dyspnea	8
Hoarseness	8
Chills	5
Leg pain	5
Low back pain	5
Shoulder pain	5
Altered mental status	4
Chest discomfort	3
Numb hands	3
Tinnitus	3
Vascular access occlusion	3
Abdominal pain	2
Hypersomnia	1
Palpitation	1
Pruritus	1

### Feature Extraction

To avoid the artifacts at the beginning of the data due to the different procedures on how the dialysis was set up and started in each HD session, the first data point Y_j,1_ at the beginning of each HD session was excluded if the blood flow rate varied between T_1_ and T_2_. We also excluded the data point Y_j,k_ when the blood flow rate was equal to or below zero due to dialysis interruption (dialyzer exchange or patient urination/defecation). An entire HD session was excluded from the analysis if the session was interrupted more than once.

In our main analysis, the whole data set {Y_j,k_, T_k_} of an HD session was included for feature extraction if no adverse event was registered for the session. On the other hand, for the HD session with adverse events, only data preceding the first adverse event were included for feature extraction, meaning the length of HD was less than 4 hours. Because the time interval between two adjacent records and the length of HD sessions vary, regression analysis is challenging, and we need to include the temporal features of the measured variables in the analyses for classification. To this end, we derived the mean, standard deviation of the mean, and coefficient of variance, as well as the slope and R square of linear regression from the dialysis and physiological measurements {Y_j,k_, T_k_}. We also derived the maximum, minimum, and mean of change rate (the first-order derivative), as well as the second-order derivative of venous pressure and transmembranous pressure as features for analysis. A total of 84 features {X_h_} (h=1-84), including those from the raw measurements {Y_j,k_, T_k_} and those derived from the temporal aspect of the data as described above, were extracted for each HD session (Multimedia Appendix 3). Feature extraction of data sets was performed using the AWK program (source code available in the format of .awk; Multimedia Appendix 4).

As aforementioned, the dialysis data set {Y_j,k_, T_k_} is recorded once the values of venous pressure or transmembranous pressure change. Therefore, the value of any measurement at the time T_p_ between two measured time stamps, T_k_ and T_k-1_, can be assigned as {Y_j,k_, T_p_} = {Y_j,k_, T_k-1_}. Thus, feature extraction of the data in an HD session could be terminated at an arbitrary time (T_p_).

### Outcome Labeling for Model Building

For outcome labeling, the HD sessions with one or more than one adverse event were labeled as 1, and HD sessions with no adverse event were labeled as 0. We also randomly relabeled 4221 HD sessions regardless of their true outcome as a negative control set while kept the same 0 to 1 ratio as the experimental set. A two-class classification model was built and evaluated by four-fold cross-validation using Azure (Microsoft Inc). At least three repeats were performed by introducing different random numbers for each model building.

### Selection for Top Performance Features

To pinpoint which features are more important than others in predicting HD adverse events, we also selected and used key features for model building and compared the results with that by a total of 84 features. The selection of key features was performed using MATLAB (MATrixLABoratory, MathWorks Inc) (source code available in the format of .m; Multimedia Appendix 5). A two-class classification model was built using ensemble random undersampling boosted trees by four-fold cross-validation. The score was given by summing up the percentages of true positives and true negatives.

The key feature selection process started with selecting the top feature according to the scores obtained from the model using a single feature from the 84 features once at a time. Next, the top two-feature combinations were selected from the two-feature combination pool, which was established by combining the top feature from the last step with each of the remaining 83 features. The two-feature combinations that resulted in scores higher than that of the top feature from the last step were kept for the next step. Likewise, the top three-feature combinations could be selected from the pool established by combining the top two-feature combinations with each of the remaining 82 features when the three-feature combinations scored higher than the two-feature combinations. We repeated this procedure until the top 20-feature combinations were selected. Features that most frequently appeared in these 20-feature combinations were defined as key features.

### Ethics Approval and Consent to Participate

This study was approved by the Institutional Review Board of National Yang-Ming University (N_105_0132) and the Institutional Review Board of Changhua Christian Hospital (CCH IRB No. 161005).

## Results

### Demographic Characteristics of the Study Participants

As of November 2017, we enrolled 108 patients. Multimedia Appendix 6 shows the baseline characteristics of the 108 patients at the beginning of the study. The mean age was 63.6 years, 60 (56%) patients were male, and the mean duration on dialysis was 7.7 years. A total of 47 (44%) patients had diabetes mellitus, 69 (64%) patients had hypertension, 11 (10%) patients had coronary artery disease, 12 (11%) patients had congestive heart failure, 7 (7%) patients had a history of stroke, and 2 (2%) patients had malignancy.

The list of intradialytic adverse events and the number of occurrences are shown in Table 1. Four HD sessions had more than three intradialytic adverse events, 19 HD sessions had three adverse events, 106 HD sessions had two adverse events, and 276 HD sessions had a single adverse event. Altogether, there were 406 HD sessions with adverse events out of 4221 total HD sessions (Multimedia Appendix 7).

### Performance of the Model for Prediction

To increase the outcome 1 to 0 ratios, wherein the session with an adverse event is labeled as 1 and the session without an adverse event is labeled as 0, we categorized the 27 adverse events listed in Table 1 into three groups. The first group was total events but excluded events of blood pressure elevation, vascular access occlusion, and vascular access thrombosis. A total of 323 HD sessions belonged to adverse event group 1. The second group included muscle cramps, and there were 138 HD sessions in this group. The third group included blood pressure elevation, and there were 108 HD sessions in this group.

#### Group 1: All Events Except Blood Pressure Elevation

**Figure 1:**
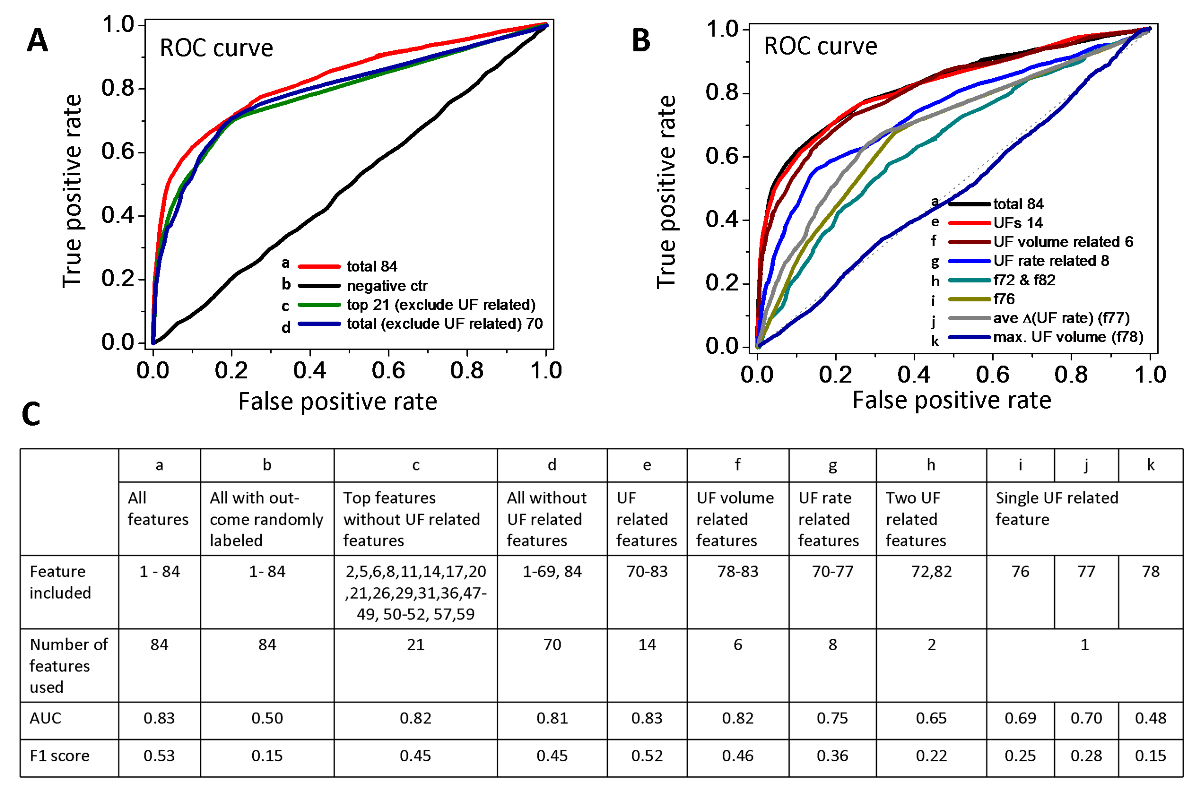
Prediction of all intradialytic adverse events except blood pressure elevation. (A) Machine learning performance represented by receiver operating characteristic (ROC) curves from 84 features (curve a, red), the top 21 features (curve c, green), all features but excluding ultrafiltration-related features (curve d, blue), and the negative control (curve b, black). Each ROC curve shown here is the average of 12 simulated ROC curves. (B). Machine learning performance represented by ROC curves is obtained from ultrafiltration-related features (curves e, f, g, h, i, j, and k) and from 84 features (curve a, black). (C) Area under the curve (AUC) and F1 scores from different feature combinations for predicting all intradialytic adverse events. UF: ultrafiltration.

A two-class averaged perceptron was used for model building with a learning rate of 20 and maximal iterations of 20. For the 84-feature model, the mean area under the curve (AUC) was 0.83 (SD 0.03), with an F1 score of 0.53, sensitivity of 0.53, and specificity of 0.96 (Figure 1A and 1B curve a). Compared with the negative control, (mean AUC=0.50, SD 0.04; F1=0.15; Figure 1A curve b), the 84-feature model of the two-class averaged perceptron could predict adverse events plausibly. Other algorithms were also tested for the prediction. The mean AUC obtained by two-class support vector machines (SVM) was 0.83 (SD 0.02), with an F1 score of 0.55, sensitivity of 0.53, and specificity of 0.96. The results were similar to those obtained by the averaged perceptron. Compared to averaged perceptron and SVM algorithms, two-class logistic regression and decision forest did not predict the adverse events well. The mean AUC obtained by logistic regression was 0.82 (SD 0.02), with an F1 score of 0.48, and the mean AUC obtained by decision forest was 0.83 (SD 0.02), with an F1 score of 0.46. Additionally, intrapatient partition and interpatient partition for sampling did not show significant difference in prediction (mean AUC=0.83, SD 0.03; mean F1=0.53, SD 0.02 vs mean AUC=0.82, SD 0.04; mean F1=0.50, SD 0.06).

Ultrafiltration rate and ultrafiltration volume are important parameters for HD. However, our models indicated that employing a single feature, such as the maximal value of ultrafiltration volume (feature 78) or the mean value of ultrafiltration rate changes (feature 77), cannot predict adverse events properly (Figure 1B curves j and k). The model built by the maximal value of ultrafiltration volume, defined as the ultrafiltration volume recorded at the last time point, had an AUC of 0.48 and F1 score of 0.15, which were similar to the results of the negative control. On the other hand, the model built by the mean value of ultrafiltration rate changes during HD sessions had an AUC of 0.70 and F1 score of 0.28. Combining two ultrafiltration-related features also failed to predict adverse events (Figure 1B curve h). After up to six ultrafiltration volume-related features (features 78-83) were used for prediction, the AUC increased from 0.48 to 0.82, and the F1 score increased from 0.15 to 0.46 (Figure 1B curve f). The model with 14 ultrafiltration features (features 70-83) had an AUC of 0.83 and F1 score of 0.52 (Figure 1B curve e).

Next, the 21 features that most frequently appeared in the 20-feature combinations were selected for the evaluation. The two-class averaged perceptron model based on these top 21 performance features but skipping ultrafiltration-related features showed a mean AUC of 0.82 (SD 0.02) and F1 score of 0.45 (Figure 1A curve c). The increase of one or two features did not enhance the prediction significantly (23 top features model: mean AUC=0.82, SD 0.02; F1=0.46). Compared with the model based on all features but excluding ultrafiltration-related features (AUC=0.81; F1=0.45; Figure 1A curve d; 70 features), the results of the 21 top features model (without ultrafiltration-related features) demonstrated that a quarter of the total 84 features was sufficient to predict adverse events.

The 21 features were age, maximum transmembranous pressure, minimum systolic blood pressure (SBP), minimum diastolic blood pressure (DBP), minimum pulse pressure, minimum blood flow rate, mean SBP, mean venous pressure, mean transmembranous pressure, slope of linear regression of SBP, slope of linear regression of DBP, slope of linear regression of pulse pressure, slope of linear regression of pulse rate, slope of linear regression of transmembranous pressure, standard deviation of the mean of blood flow rate, R-squared of linear regression of pulse pressure, and related parameters to the second-order derivative of venous pressure (features 2, 5, 6, 8, 11, 14, 17, 20, 21, 26, 29, 31, 36, 47-52, 57, and 59) (Multimedia Appendix 3).

#### Group 2: Muscle Cramps

**Figure 2:**
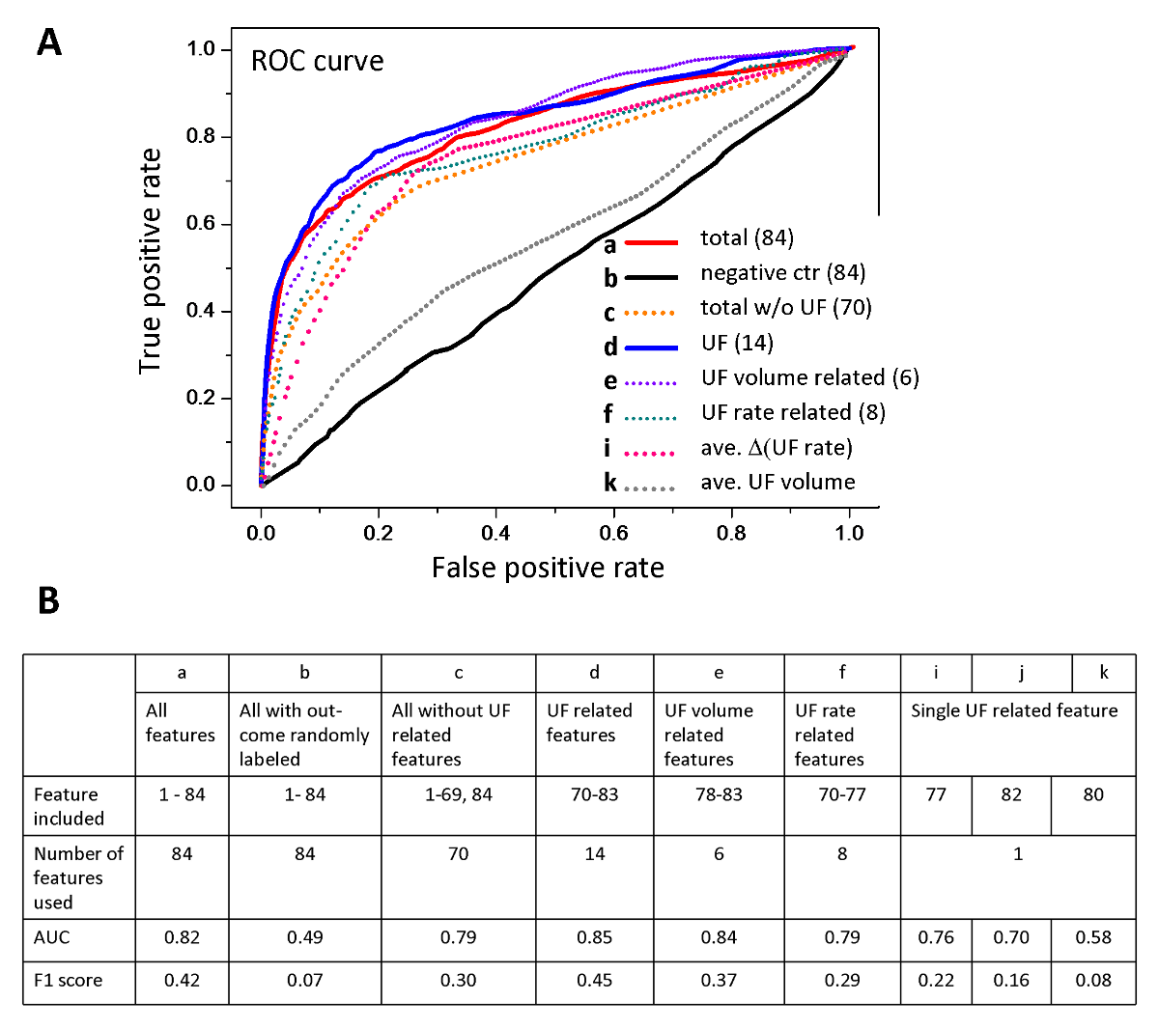
Prediction of a specific intradialytic adverse event: muscle cramps. (A) Machine learning performance is represented by receiver operating characteristic (ROC) curves from 84 features (curve a, red), all features but excluding ultrafiltration-related features (curve c, orange dot), ultrafiltration-related features (curves d, e, f, i, k), and the negative control (curve b, black). (B) Area under the curve (AUC) and F1 scores from different feature combinations for predicting muscle cramps. UF: ultrafiltration.

The model, which was based on 14 ultrafiltration-related features, had a mean AUC of 0.85 (SD 0.04) and F1 score of 0.45 (Figure 2 curve d) for predicting the occurrence of muscle cramps, and the result is similar to that of the 84-feature model (mean AUC=0.83, SD 0.04; F1=0.42; Figure 2 curve a) and better than that of the model built based on all features but excluding ultrafiltration-related features (mean AUC=0.79, SD 0.04; F1=0.30; Figure 2 curve c). However, a single ultrafiltration-related feature cannot predict cramps properly (Figure 2 curves i and k). The combination of two ultrafiltration-related features also failed to predict muscle cramps (AUC=0.79 and F1=0.29 for features 70 and 77; AUC=0.84 and F1=0.37 for features 78 and 83). Our results demonstrated that ultrafiltration-related features contribute more than other features to the prediction of muscle cramps.

#### Group 3: Blood Pressure Elevation

**Figure 3:**
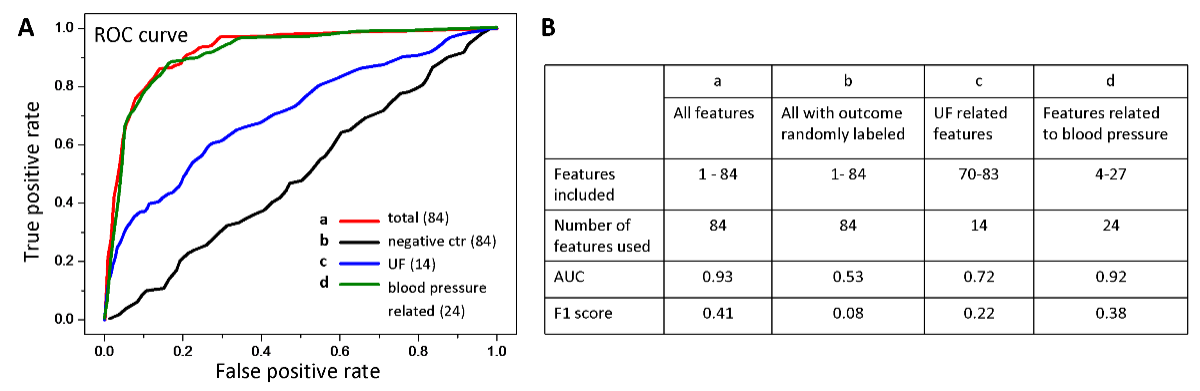
Prediction of a specific intradialytic adverse event: blood pressure elevation. (A) Machine learning performance represented by receiver operating characteristic (ROC) curves from 84 features (curve a, red), blood pressure-related features (curve d, green), ultrafiltration-related features (curve c, blue), and the negative control (curve b, black). (B) Area under the curve (AUC) and F1 scores from different feature combinations for predicting blood pressure elevation. UF: ultrafiltration.

The model, which was based on a total of 84 features, had a mean AUC of 0.93 (SD 0.02) and F1 score of 0.41 for predicting the occurrence of hypertension (Figure 3 curve a). Compared with the model built based on 14 ultrafiltration-related features (AUC=0.72; F1=0.22; Figure 3 curve c), our results demonstrated that ultrafiltration parameters did not play important roles in predicting intradialytic hypertension. Even though the model based on 24 blood pressure–related features had an AUC higher than 0.9 (AUC=0.92, SD 0.03; F1=0.38; Figure 3 curve d), features other than blood pressure can further contribute to an additional improvement in the F1 score.

### Consistency of the Predicted Probabilities of Adverse Events Over Time

**Figure 4:**
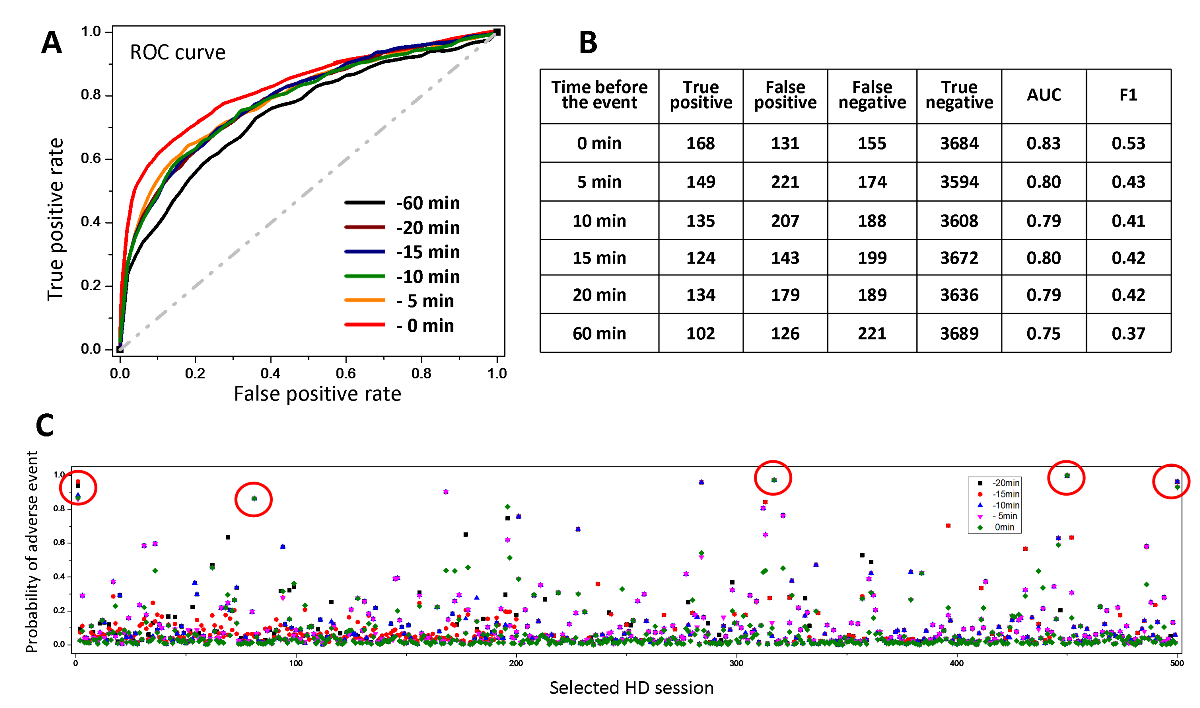
Prediction performance for group 1 intradialytic adverse events using the features of different cutoff ending time points. (A) Machine learning performance represented by receiver operating characteristic (ROC) curves from 84 features extracted from [Yj, Tk]HD terminated at different cutoff ending time points as follows: one time point before an adverse event (noted as 0 minutes), and 5, 10, 15, 20, and 60 minutes before an adverse event or before the end of the hemodialysis (HD) session if no adverse event. (B) Area under the curve (AUC) and F1 scores. (C) Probability of adverse event occurrence in 500 randomly selected HD sessions. The red circle indicates the HD session with adverse events, and the predicted probabilities of adverse events are all higher than 0.8 independent of the cutoff ending time point.

As shown in Figure 1, the time series features were collected throughout the HD session, from the beginning of HD to the time point right before the index adverse event or right before the end of the HD session, which has no adverse event. In this case, we defined the end of feature collection as 0 minutes if this ending time point was right before the occurrence of the index adverse event. In addition to 0 minutes, we also set the cutoff ending time points of feature collection as 5, 10, 15, 20, and 60 minutes before the occurrence of the adverse event to evaluate the prediction accuracy. Machine learning for predicting all intradialytic adverse events, except blood pressure elevation, showed that features of the 0-minute cutoff led to the best AUC and F1 score (AUC=0.83; F1=0.53) compared to those learned from the features of earlier cutoff time points (Figure 4A and 4B), even though AUC scores from the features of the cutoff ending time points 5, 10, 15, and 20 minutes before the index adverse event or before the end of HD sessions without an adverse event were about 0.80 and their F1 scores were all lower than 0.5. The results suggest that while the information embedded in the 20-minute time window before the index adverse event is valuable, the information embedded in the 5-minute time window before the index adverse event is more influential for event prediction.

To further understand the cutoff ending time point dependence of prediction accuracy, 500 HD sessions were randomly selected to compare the prediction probabilities of adverse events obtained from 84 features with cutoff ending time points of 0, 5, 10, 15, and 20 minutes before the index adverse event. As shown in Figure 4C (red circles), five HD sessions possessed strong consistency in the predicted probabilities of adverse events using extracted features based on different cutoff ending time points, and adverse events indeed occurred in these five HD sessions. Since there should be approximately 40 HD sessions developing adverse events among 500 randomly selected HD sessions, the results suggest that at least one-tenth of HD sessions with adverse events can be sighted as early as 20 minutes in advance and can be further confirmed by real-time machine learning using features from subsequent cutoff ending time points (Figure 4C).

Even though none of the 84 features contained explicit time series information, the linear and differential analyses that feature extraction employed may be affected by the length of HD sessions. Therefore, we truncated the HD sessions with no adverse events (negative ones) and compared the prediction results with those from the untruncated ones. Since the average length of HD sessions with adverse events (positive ones) was 3.3 hours, negative HD sessions were truncated and randomly assigned endpoints (T_end_) between 3 and 3.5 hours, yet the endpoints of positive ones remained unchanged. The data set {Y_j,k_, T_k_} at endpoint T_end_ was defined according to the same method used for {Y_j,k_, T_k_} at arbitrary time T_p_. Regarding the results, the mean AUC was 0.89 (SD 0.019), F1 score was 0.55, sensitivity was 0.52, and specificity was 0.97. Alternately, the AUC was 0.86 with an F1 score of 0.55 when the endpoints were assigned exactly at 3.3 hours. Compared to the original results obtained from the untruncated negative HD sessions with a duration of about 4 hours (AUC=0.83, F1=0.53, sensitivity=0.53, and specificity=0.96), the prediction results were better when the endpoints were set earlier. Indeed, the AUC was 0.92, with an F1 score of 0.62, sensitivity of 0.61, and specificity of 0.98, when the endpoints were randomly assigned between 2.5 and 3.5 hours for negative HD sessions.

## Discussion

### Contributions and Principal Findings

Our findings indicate that algorithms combining linear and differential analyses with two-class classification machine learning predict intradialytic adverse events with high AUCs. We attempted to identify features that contribute the most to predicting all adverse events, except hypertension, (group 1) from a total of 84 features extracted from [Y_j_, T_k_]_HD_. Among the top 23 features, only feature 76 and feature 82 were related to ultrafiltration (the number of times that the ultrafiltration rate changes and the linear regression slope of ultrafiltration volume). After excluding these two ultrafiltration-related features, we found that the remaining 21 features were sufficient for accurate prediction with good discriminating power, with a slight reduction in the AUC from 0.83 (84 features) to 0.82 (21 features). The model built by 14 ultrafiltration-related features also had a good AUC of 0.83. Therefore, instead of including all 84 features for model building, selecting the top 21 ultrafiltration-unrelated features or integrating a total of 14 ultrafiltration-related features can reduce computing load. Our results also suggest that these two clusters of features (Figure 1A curve c and Figure 1B curve e) may embed similar factors contributing to the onset of adverse events.

In our study, muscle cramp was an adverse event that occurred most frequently during HD treatment. A muscle cramp is a common adverse event that happens during HD therapy, with a prevalence of 28% among all HD sessions [5,34]. Muscle cramps result from ischemia of the skeletal muscle tissue, indicating an early sign of hypotension, and it may lead to premature discontinuation of HD sessions [35,36]. Tissue ischemia during HD is positively related to the ultrafiltration rate [5,37,38]. Indeed, the model built by 14 ultrafiltration-related features had an AUC of 0.85 for predicting the occurrence of muscle cramps in this study. We excluded all ultrafiltration-related features to test the prediction accuracy, including ultrafiltration rate and ultrafiltration volume, and the AUC reduced from 0.82 (84 features) to 0.79 (70 features), indicating that ultrafiltration-related features are important but not necessarily required to predict muscle cramps. The results from machine learning revealed that ultrafiltration-independent features contribute to predicting intradialytic muscle cramps as well.

In general, symptomatic hypotension occurs in 20% to 30% of dialysis sessions [6,39-43]. There are two major pathophysiological mechanisms of intradialytic hypotension. First, when plasma fluid removal through ultrafiltration exceeds the rate of plasma refilling into the blood vessels, blood volume reduces [5]. In the meantime, if the cardiovascular and neurohormone systems fail to compensate for the acute vascular volume depletion during ultrafiltration, hypotension occurs [3,44-47]. Frequent episodes of intradialytic hypotension may cause reduced ultrafiltration, inadequate “dry weight,” increased preload, and impaired heart function that eventually leads to more episodes of hypotension, thus creating a vicious cycle. Meanwhile, frequent intradialytic hypotension disrupts dialysis efficiency and efficacy. It is associated with higher morbidity and mortality [48-51], which partly contributes to the fact that cardiovascular disease is the leading cause of morbidity and mortality in HD patients [52]. Lin et al recently developed an intelligent system to predict intradialytic hypotension [53]. However, our prediction models not only further precluded ultrafiltration-related features but also examined overall intradialytic adverse events instead of only focusing on the hypotensive episode.

As shown in Multimedia Appendix 8, the top 16 features that majorly contributed to predicting muscle cramps included patient characteristics, venous pressure, transmembranous pressure, ultrafiltration, blood flow rate, and pulse pressure. The minimal value of venous pressure and the mean value of transmembranous pressure were features that had the most hits (20 and 17 hits, respectively). These top two features for predicting muscle cramps were derived from dialysis machine output parameters, indicating that there is a potential to integrate our algorithm into the dialysis machine software to alert clinicians and to adjust dialysis machine settings in advance. Nevertheless, unlike prediction of all adverse events, for which only two out of the top 23 features were related to ultrafiltration, eight out of the top 16 features were related to ultrafiltration in terms of predicting muscle cramps, indicating that ultrafiltration-related parameters are important factors of muscle cramps.

Compared with several two-class classification modules, such as Bayes point machine, boosted decision tree, and SVM, models built by two-class average perceptron had the best AUC and F1 score. We also built models by deep learning (data not shown), but the results from deep learning did not show a good AUC and F1 score, possibly due to the limited number of our HD data sets. As clinicians are now facing the new era of artificial intelligence [54], the integration of computer science and dialysis medicine could be regarded as the first step to improve HD patients’ care quality comprehensively. Our study demonstrated the feasibility of this integration. Even though the limited number of data sets and imbalanced data outcomes in our research hinder better prediction accuracy, it is anticipated that increased data sets will further improve the AUC and F1 score. Moreover, integrating machine learning with the dialysis machine and modifying algorithms in real-time by cloud computing with accumulation of data sets could enhance prediction performance.

Several questions may be answered if the size of the HD data set is expanded in future studies. First, how early can we predict adverse events? The consistency in the predicted probabilities of adverse events using features based on different cutoff ending time points could detect about one-tenth of HD sessions with adverse events (Figure 4). We anticipate that an increase in the number of HD sessions with adverse events for model training can improve imbalanced data and possibly bring forward the timing for the alert. Second, since most of the adverse events took place in second-half HD sessions, whether the data sets of second-half HD sessions are sufficient for prediction can be further studied. Finally, if more HD sessions with adverse events are recruited, we can build models for different adverse events instead of grouping the events to reduce imbalanced data outcomes.

### Conclusion

In this study, a model of two-class classification was established to predict intradialytic adverse events in quasi-real time, with AUCs higher than 0.8. The consistency in the predicted probabilities of adverse events obtained from the features extracted in the ongoing HD process in real time could have the HD session tagged for forthcoming adverse events. Such a methodology implemented with local cloud computation could warn clinicians to take necessary actions and adjust the HD machine settings in advance.

## Appendix

Multimedia Appendix 1 List of hemodialysis machine readouts.

Multimedia Appendix 2 An example of physiological and dialysis data collected in one hemodialysis (HD) session.

Multimedia Appendix 3 List of 84 features.

Multimedia Appendix 4 Source code: Feature extraction.

Multimedia Appendix 5 Source code: Top performance feature selection.

Multimedia Appendix 6 Demographic characteristics of the study participants (n=108).

Multimedia Appendix 7 Simple decision tree of hemodialysis (HD) patients and HD sessions used for the study. One hundred twenty-nine patients were eligible after the enrollment evaluation, and 108 patients completed the 3-month study. There were 4221 HD sessions used for model building, and of these, 3815 did not have adverse events and 406 had one or multiple adverse events.

Multimedia Appendix 8 Top 16 features for predicting intradialytic muscle cramps.

## Abbreviations

AUC: area under the curve
DBP: diastolic blood pressure
HD: hemodialysis
SBP: systolic blood pressure
SVM: support vector machines

## References

[1] Ricci Z, Romagnoli S (2014). Renal replacement therapy for critically ill patients: an intermittent continuity. Crit Care.

[2] Douvris A, Malhi G, Hiremath S, McIntyre L, Silver SA, Bagshaw SM, Wald R, Ronco C, Sikora L, Weber C, Clark EG (2018). Interventions to prevent hemodynamic instability during renal replacement therapy in critically ill patients: a systematic review. Crit Care.

[3] Daugirdas JT (2001). Pathophysiology of dialysis hypotension: an update. Am J Kidney Dis.

[4] Garzoni D, Keusch G, Kleinoeder T, Martin H, Dhondt A, Cremaschi L, Tatsis E, Ibrahim N, Boer W, Kuehne S, Claus M, Zahn M, Schuemann E, Engelmann J, Hickstein H, Wojke R, Gauly A, Passlick-Deetjen J (2007). Reduced complications during hemodialysis by automatic blood volume controlled ultrafiltration. Int J Artif Organs.

[5] Steuer RR, Leypoldt JK, Cheung AK, Harris DH, Conis JM (1994). Hematocrit as an indicator of blood volume and a predictor of intradialytic morbid events. ASAIO J.

[6] Daugirdas JT (1994). Preventing and Managing Hypotension. Seminars in Dialysis.

[7] Santoro A, Mancini E, Basile C, Amoroso L, Di Giulio S, Usberti M, Colasanti G, Verzetti G, Rocco A, Imbasciati E, Panzetta G, Bolzani R, Grandi F, Polacchini M (2002). Blood volume controlled hemodialysis in hypotension-prone patients: a randomized, multicenter controlled trial. Kidney Int.

[8] Donauer J, Kölblin D, Bek M, Krause A, Böhler J (2000). Ultrafiltration profiling and measurement of relative blood volume as strategies to reduce hemodialysis-related side effects. Am J Kidney Dis.

[9] Shoji T, Tsubakihara Y, Fujii M, Imai E (2004). Hemodialysis-associated hypotension as an independent risk factor for two-year mortality in hemodialysis patients. Kidney Int.

[10] Stefánsson BV, Brunelli SM, Cabrera C, Rosenbaum D, Anum E, Ramakrishnan K, Jensen DE, Stålhammar NO (2014). Intradialytic hypotension and risk of cardiovascular disease. Clin J Am Soc Nephrol.

[11] Steuer RR, Harris DH, Weiss RL, Biddulph MC, Conis JM (1991). Evaluation of a noninvasive hematocrit monitor: a new technology. Am Clin Lab.

[12] Steuer RR, Leypoldt JK, Cheung AK, Senekjian HO, Conis JM (1996). Reducing symptoms during hemodialysis by continuously monitoring the hematocrit. American Journal of Kidney Diseases.

[13] Sinha AD, Light RP, Agarwal R (2010). Relative plasma volume monitoring during hemodialysis AIDS the assessment of dry weight. Hypertension.

[14] Rodriguez HJ, Domenici R, Diroll A, Goykhman I (2005). Assessment of dry weight by monitoring changes in blood volume during hemodialysis using Crit-Line. Kidney Int.

[15] Reddan DN, Szczech LA, Hasselblad V, Lowrie EG, Lindsay RM, Himmelfarb J, Toto RD, Stivelman J, Winchester JF, Zillman LA, Califf RM, Owen WF (2005). Intradialytic blood volume monitoring in ambulatory hemodialysis patients: a randomized trial. J Am Soc Nephrol.

[16] Akl AI, Sobh MA, Enab YM, Tattersall J (2001). Artificial intelligence: a new approach for prescription and monitoring of hemodialysis therapy. Am J Kidney Dis.

[17] Gabutti L, Vadilonga D, Mombelli G, Burnier M, Marone C (2004). Artificial neural networks improve the prediction of Kt/V, follow-up dietary protein intake and hypotension risk in haemodialysis patients. Nephrol Dial Transplant.

[18] Goldfarb-Rumyantzev A, Schwenk MH, Liu S, Charytan C, Spinowitz BS (2003). Prediction of single-pool Kt/v based on clinical and hemodialysis variables using multilinear regression, tree-based modeling, and artificial neural networks. Artif Organs.

[19] Guh JY, Yang CY, Yang JM, Chen LM, Lai YH (1998). Prediction of equilibrated postdialysis BUN by an artificial neural network in high-efficiency hemodialysis. Am J Kidney Dis.

[20] Gabutti L, Burnier M, Mombelli G, Malé F, Pellegrini L, Marone C (2004). Usefulness of artificial neural networks to predict follow-up dietary protein intake in hemodialysis patients. Kidney Int.

[21] Chiu J, Chong C, Lin Y, Wu C, Wang Y, Li Y (2005). Applying an artificial neural network to predict total body water in hemodialysis patients. Am J Nephrol.

[22] Barbieri C, Bolzoni E, Mari F, Cattinelli I, Bellocchio F, Martin JD, Amato C, Stopper A, Gatti E, Macdougall IC, Stuard S, Canaud B (2016). Performance of a Predictive Model for Long-Term Hemoglobin Response to Darbepoetin and Iron Administration in a Large Cohort of Hemodialysis Patients. PLoS One.

[23] Barbieri C, Molina M, Ponce P, Tothova M, Cattinelli I, Ion Titapiccolo J, Mari F, Amato C, Leipold F, Wehmeyer W, Stuard S, Stopper A, Canaud B (2016). An international observational study suggests that artificial intelligence for clinical decision support optimizes anemia management in hemodialysis patients. Kidney Int.

[24] Barbieri C, Mari F, Stopper A, Gatti E, Escandell-Montero P, Martínez-Martínez JM, Martín-Guerrero JD (2015). A new machine learning approach for predicting the response to anemia treatment in a large cohort of End Stage Renal Disease patients undergoing dialysis. Comput Biol Med.

[25] Escandell-Montero P, Chermisi M, Martínez-Martínez JM, Gómez-Sanchis J, Barbieri C, Soria-Olivas E, Mari F, Vila-Francés J, Stopper A, Gatti E, Martín-Guerrero JD (2014). Optimization of anemia treatment in hemodialysis patients via reinforcement learning. Artif Intell Med.

[26] Gabutti L, Lötscher N, Bianda J, Marone C, Mombelli G, Burnier M (2006). Would artificial neural networks implemented in clinical wards help nephrologists in predicting epoetin responsiveness?. BMC Nephrol.

[27] Martínez-Martínez JM, Escandell-Montero P, Barbieri C, Soria-Olivas E, Mari F, Martínez-Sober M, Amato C, Serrano López AJ, Bassi M, Magdalena-Benedito R, Stopper A, Martín-Guerrero JD, Gatti E (2014). Prediction of the hemoglobin level in hemodialysis patients using machine learning techniques. Comput Methods Programs Biomed.

[28] Saadat S, Aziz A, Ahmad H, Imtiaz H, Sohail ZS, Kazmi A, Aslam S, Naqvi N, Saadat S (2017). Predicting Quality of Life Changes in Hemodialysis Patients Using Machine Learning: Generation of an Early Warning System. Cureus.

[29] Cattinelli I, Bolzoni E, Chermisi M, Bellocchio F, Barbieri C, Mari F, Amato C, Menzer M, Stopper A, Gatti E (2013). Computational intelligence for the Balanced Scorecard: studying performance trends of hemodialysis clinics. Artif Intell Med.

[30] Bellazzi R, Larizza C, Magni P, Bellazzi R (2005). Temporal data mining for the quality assessment of hemodialysis services. Artif Intell Med.

[31] Guo Y, Hao Z, Zhao S, Gong J, Yang F (2020). Artificial Intelligence in Health Care: Bibliometric Analysis. J Med Internet Res.

[32] Sheng K, Zhang P, Yao X, Li J, He Y, Chen J (2020). Prognostic Machine Learning Models for First-Year Mortality in Incident Hemodialysis Patients: Development and Validation Study. JMIR Med Inform.

[33] Gabutti L, Machacek M, Marone C, Ferrari P (2005). Predicting intradialytic hypotension from experience, statistical models and artificial neural networks. J Nephrol.

[34] Sherman RA, Goodling KA, Eisinger RP (1982). Acute Therapy of Hemodialysis-Related Muscle Cramps. American Journal of Kidney Diseases.

[35] Jenkins PG, Dreher WH (1975). Dialysis-induced muscle cramps: treatment with hypertonic saline and theory as to etiology. Trans Am Soc Artif Intern Organs.

[36] Rocco MV, Burkart JM (1993). Prevalence of missed treatments and early sign-offs in hemodialysis patients. J Am Soc Nephrol.

[37] de Vries PM, Kouw PM, Olthof CG, Solf A, Schuenemann B, Oe LP, Quellhorst E, Donker AJ (1990). The influence of dialysate sodium and variable ultrafiltration on fluid balance during hemodialysis. ASAIO Trans.

[38] Bogaard HJ, de Vries JP, de Vries PM (1994). Assessment of refill and hypovolaemia by continuous surveillance of blood volume and extracellular fluid volume. Nephrol Dial Transplant.

[39] Zucchelli P, Santoro A (1993). Dialysis-induced hypotension: a fresh look at pathophysiology. Blood Purif.

[40] Orofino L, Marcén R, Quereda C, Villafruela JJ, Sabater J, Matesanz R, Pascual J, Ortuño J (1990). Epidemiology of symptomatic hypotension in hemodialysis: is cool dialysate beneficial for all patients?. Am J Nephrol.

[41] Degoulet P, Réach I, Di Giulio S, Devriès C, Rouby JJ, Aimé F, Vonlanthen M (1981). Epidemiology of dialysis induced hypotension. Proc Eur Dial Transplant Assoc.

[42] Chen I, Chang M, Chiao S, Chen J, Yu C, Yang S, Liu J, Hung C, Yang R, Chang H, Hsu C, Fang J (2012). Korean red ginseng improves blood pressure stability in patients with intradialytic hypotension. Evid Based Complement Alternat Med.

[43] Palmer BF, Henrich WL (2008). Recent advances in the prevention and management of intradialytic hypotension. J Am Soc Nephrol.

[44] Yamamoto K, Kobayashi N, Kutsuna T, Ishii A, Matsumoto T, Hara M, Aiba N, Tabata M, Takahira N, Masuda T (2012). Excessive fall of blood pressure during maintenance hemodialysis in patients with chronic renal failure is induced by vascular malfunction and imbalance of autonomic nervous activity. Ther Apher Dial.

[45] Perazella MA, Rho M, Brewster UC (2008). Vasopressin insufficiency and intradialytic hypotension. Am J Kidney Dis.

[46] Coen G, Mantella D, Sardella D, Beraldi MP, Ferrari I, Pierantozzi A, Lippi B, Di Giulio S (2009). Asymmetric dimethylarginine, vascular calcifications and parathyroid hormone serum levels in hemodialysis patients. J Nephrol.

[47] Rashid G, Bernheim J, Green J, Benchetrit S (2007). Parathyroid hormone stimulates endothelial expression of atherosclerotic parameters through protein kinase pathways. Am J Physiol Renal Physiol.

[48] Henderson LW (2012). Symptomatic intradialytic hypotension and mortality: an opinionated review. Semin Dial.

[49] Inrig JK, Oddone EZ, Hasselblad V, Gillespie B, Patel UD, Reddan D, Toto R, Himmelfarb J, Winchester JF, Stivelman J, Lindsay RM, Szczech LA (2007). Association of intradialytic blood pressure changes with hospitalization and mortality rates in prevalent ESRD patients. Kidney Int.

[50] Tislér A, Akócsi K, Borbás B, Fazakas L, Ferenczi S, Görögh S, Kulcsár I, Nagy L, Sámik J, Szegedi J, Tóth E, Wágner G, Kiss I (2003). The effect of frequent or occasional dialysis-associated hypotension on survival of patients on maintenance haemodialysis. Nephrol Dial Transplant.

[51] Chou JA, Streja E, Nguyen DV, Rhee CM, Obi Y, Inrig JK, Amin A, Kovesdy CP, Sim JJ, Kalantar-Zadeh K (2018). Intradialytic hypotension, blood pressure changes and mortality risk in incident hemodialysis patients. Nephrol Dial Transplant.

[52] No authors listed (1997). Causes of death. USRDS. United States Renal Data System. Am J Kidney Dis.

[53] Lin C, Chen C, Wu P, Pan C, Shih H, Huang M, Chou L, Tang J, Wu C (2018). Intelligent system to predict intradialytic hypotension in chronic hemodialysis. J Formos Med Assoc.

[54] Lemley KV (2019). Machine Learning Comes to Nephrology. JASN.

