# Supplementary material for: Machine Learning Analysis of Time-Dependent Features for Predicting Adverse Events During Hemodialysis Therapy: Model Development and Validation Study
Source: J Med Internet Res. 2021 Sep 7;23(9):e27098. doi: 10.2196/27098 (PMC8456349; doi:10.2196/27098)
Supplement: Multimedia Appendix 1 [file jmir_v23i9e27098_app1.doc]

| **Multimedia Appendix 1.** List of hemodialysis machine readouts. | | |
| --- | --- | --- |
| Parameter | Unit | Abbreviation |
| Systolic blood pressure | mmHg | SBP |
| Diastolic blood pressure | mmHg | DBP |
| Pulse rate | beat/min | PR |
| Blood flow | mL/min | BF |
| Venous pressure | mmHg | VP |
| Transmembranous pressure | mmHg | TMP |
| Dialysate conductivity | % |  |
| Dialysate temperature | °C |  |
| Ultrafiltration rate | L/hour | UF rate |
| Ultrafiltration volume | L | UF volume |
|  |  |  |
