# Supplementary figures and images for: Machine Learning Analysis of Time-Dependent Features for Predicting Adverse Events During Hemodialysis Therapy: Model Development and Validation Study
Source: J Med Internet Res. 2021 Sep 7;23(9):e27098. doi: 10.2196/27098 (PMC8456349; doi:10.2196/27098)

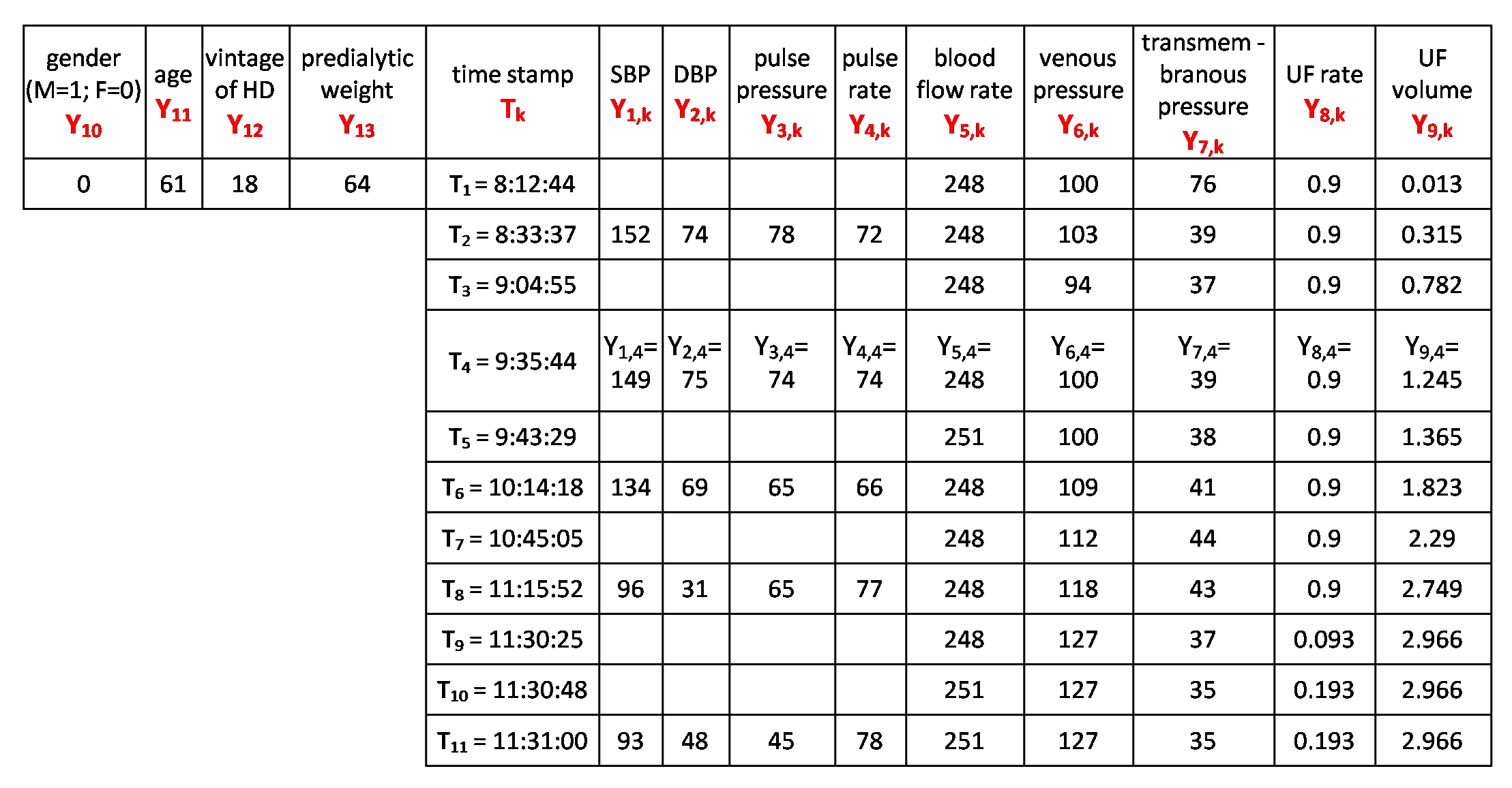

Supplement: Multimedia Appendix 2 [file jmir_v23i9e27098_app2.png]

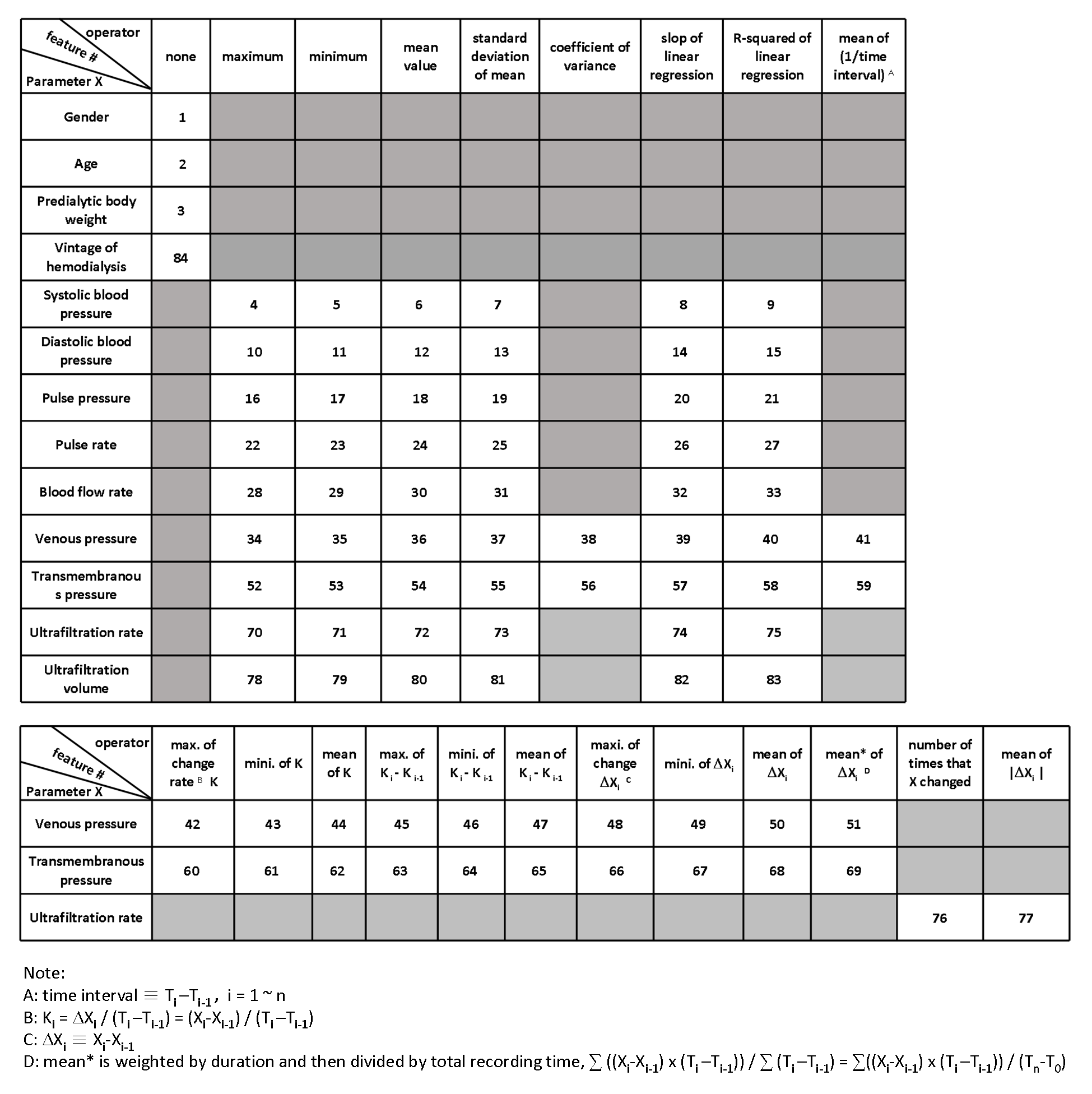

Supplement: Multimedia Appendix 3 [file jmir_v23i9e27098_app3.png]

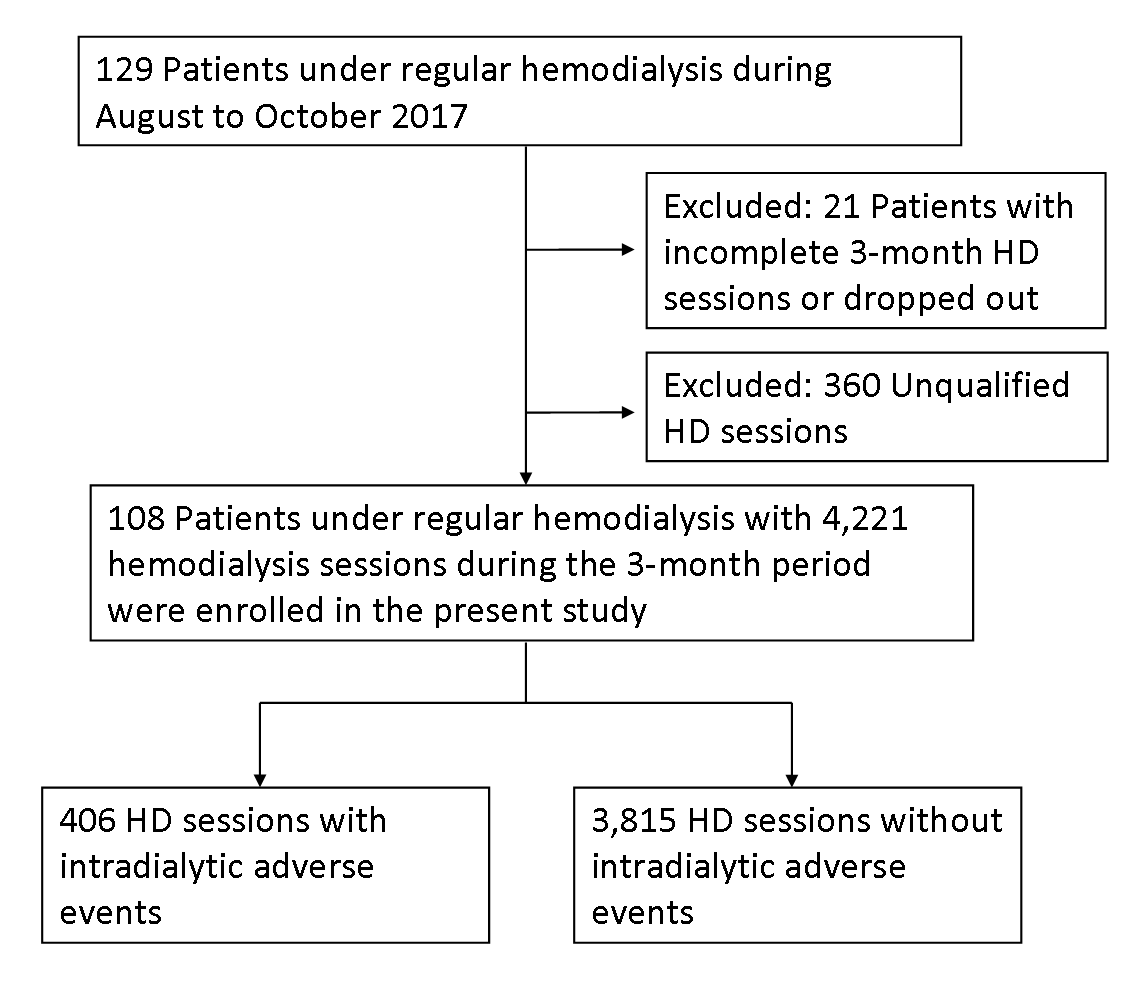

Supplement: Multimedia Appendix 7 [file jmir_v23i9e27098_app7.png]
