# Supplementary material for: Machine Learning Analysis of Time-Dependent Features for Predicting Adverse Events During Hemodialysis Therapy: Model Development and Validation Study
Source: J Med Internet Res. 2021 Sep 7;23(9):e27098. doi: 10.2196/27098 (PMC8456349; doi:10.2196/27098)
Supplement: Multimedia Appendix 6 [file jmir_v23i9e27098_app6.doc]

| **Multimedia Appendix 6.** Demographic characteristics of the study participants (*n*=108). | | |
| --- | --- | --- |
| Age (year) | | 63.6 ± 11.1 |
| Male gender (*n*; %) | | 60; 55.6 |
| Dialysis vintage (year) | | 7.7 ± 6.2 |
| Comorbidities | |  |
|  | Diabetes mellitus (*n*; %) | 47; 43.5 |
|  | Hypertension (*n*; %) | 69; 63.9 |
|  | Coronary artery disease (*n*; %) | 11; 10.2 |
|  | Congestive heart failure (*n*; %) | 12; 11.1 |
|  | Prior stroke (*n*; %) | 7; 6.5 |
|  | Chronic obstructive pulmonary disease (*n*; %) | 3; 2.8 |
|  | Peripheral vascular disease (*n*; %) | 2; 1.9 |
|  | Malignancy (*n*; %) | 2; 1.9 |
| Data were presented as mean ± SD or percentage as appropriate. | | |
