# Supplementary material for: Machine Learning Analysis of Time-Dependent Features for Predicting Adverse Events During Hemodialysis Therapy: Model Development and Validation Study
Source: J Med Internet Res. 2021 Sep 7;23(9):e27098. doi: 10.2196/27098 (PMC8456349; doi:10.2196/27098)
Supplement: Multimedia Appendix 8 [file jmir_v23i9e27098_app8.doc]

| **Multimedia Appendix 8.** Top 16 features for predicting intradialytic muscle cramps. | | | |
| --- | --- | --- | --- |
| Top | Hits | Feature# | Feature |
| 1 | 20 | 35 | Venous pressure-minimum |
| 2 | 17 | 54 | Transmembranous pressure-mean |
| 3 | 15 | 2 | Age |
| 4 | 14 | 84 | The vintage of hemodialysis |
| 5 | 13 | 77 | Ultrafiltration rate change value-mean |
| 6 | 12 | 73 | Ultrafiltration rate change-standard deviation |
| 7 | 12 | 81 | Ultrafiltration volume-standard deviation |
| 8 | 11 | 30 | Blood flow rate-mean |
| 9 | 11 | 82 | Ultrafiltration volume linear regression slope |
| 10 | 10 | 72 | Ultrafiltration rate-mean |
| 11 | 10 | 76 | Ultrafiltration rate-times of change |
| 12 | 9 | 79 | Ultrafiltration volume-minimum |
| 13 | 9 | 80 | Ultrafiltration volume-mean |
| 14 | 8 | 16 | Pulse pressure-maximum |
| 15 | 8 | 19 | Pulse pressure-standard deviation |
| 16 | 8 | 28 | Blood flow rate-maximum |
|  | | | |
